# Association between HPV infection and prostate cancer in a Mexican population

**DOI:** 10.1590/1678-4685-GMB-2017-0331

**Published:** 2018-11-29

**Authors:** Olivia Medel-Flores, Vania Alejandra Valenzuela-Rodríguez, Rodolfo Ocadiz-Delgado, Leonardo Josué Castro-Muñoz, Sandra Hernández-Leyva, Gabriel Lara-Hernández, Jesús-Gabriel Silva-Escobedo, Patricio Gariglio Vidal, Virginia Sánchez-Monroy

**Affiliations:** ^1^ Laboratorio de Biomedicina Molecular I, Escuela Nacional de Medicina y Homeopatía, Instituto Politécnico Nacional, Ciudad de México, Mexico; ^2^ Laboratorio Multidisciplinario de Investigación, Escuela Militar de Graduados de Sanidad, Secretaría de la Defensa Nacional, Ciudad de México, Mexico; ^3^ Departamento de Genética y Biología Molecular, Centro de Investigación y de Estudios Avanzados del Instituto Politécnico Nacional, Ciudad de México, Mexico

**Keywords:** HPV, prostate, cancer, koilocytes

## Abstract

The aim of this study was to evaluate the association between prostate cancer (PCa) and Human papillomavirus (HPV) infection in the Mexican population. We studied 356 paraffin-embedded tissues from unrelated Mexican men with PCa or benign prostatic hyperplasia (BPH), with the latter serving as control. HPV detection was performed by polymerase chain reaction (PCR) using universal primers, and viral genotypes were detected using sequencing or multiplex PCR. Light microscopy analyses enabled the identification of koilocytes in samples subsequently analyzed for HPV detection by *in situ* PCR and for p16-INK4A expression by immunohistochemistry. The results showed that high risk- (HR) HPVs were detected in 37/189 (19.6%) PCa specimens compared to 16/167 (9.6%) of BHP specimens (odds ratio 2.3; 95% CI= 1.2 to 4.3; *p*=0.01). These data suggest HR-HPV may play a role in PCa. HPV 52 and 58 were the most frequent genotypes (33 and 17%, respectively) detected in the population studied. Koilocytes were detected in all *in situ* PCR-HPV-positive samples, representing a pathognomonic feature of infection, and we observed the overexpression of p16-INK4A in HPV-positive samples compared to HPV-negative samples, indirectly suggesting the presence of HR-HPV E7 oncoprotein. These results suggest that HPV infection plays an important role in prostate cancer development.

## Introduction

Prostate cancer (PCa) is the second most common cancer and the fifth leading cause of death from cancer in men (Ferlay *et al.*, 2012). Infectious agents represent a risk factor in cancer pathogenesis (Chen *et al.*, 2014). Clinical and epidemiological evidence has demonstrated that infections may lead to chronic inflammation, which induces an inflammatory microenvironment that promotes the proliferation and survival of malignant cells, angiogenesis and metastasis, subverts adaptive immune responses, and alters responses to hormones and chemotherapeutic agents (Coussens and Werb, 2002; Mantovani *et al.*, 2008).

Human papillomavirus (HPV) infection is one of the most common sexually transmitted infections (STIs) worldwide (Heidegger *et al.*, 2015). Based on the findings of epidemiological and mechanistic studies, HPV types 16, 18, 31, 33, 35, 39, 45, 51, 52, 56, 58, and 59 have been classified by the International Agency for Research on Cancer (IARC) as human carcinogens (Chen *et al.*, 2014). High-risk (HR) genotypes of HPV cause cancer, particularly cervical, anal, vulvar/vaginal, penile, and oropharyngeal (Gillison *et al.*, 2015; Gao and Smith, 2016; Stratton and Culkin, 2016; Nelson and Benson, 2017).

HPV infection is also one of the causes of intraprostatic inflammation, and there is evidence showing that chronic inflammation is involved in the regulation of cellular events in prostate carcinogenesis (Jiang *et al.*, 2013; Sfanos *et al.*, 2013; Caini *et al.*, 2014; Taverna *et al.*, 2015).

A recent meta-analysis of 26 tissue-based case-control studies showed a significantly increased risk of PCa in the presence of HPV infection (Yang *et al.*, 2015). In México, the association between the detection of HPV DNA in prostatic tissue and the frequency of viral genotypes has been poorly investigated (Martinez-Fierro *et al.*, 2010; Dávila-Rodríguez *et al.*, 2016), and mainly cervical tissues have been studied as is summarized in Table 1. The present study examined the association of HPV detection and viral genotypes in prostate carcinomas in Mexican men.

**Table 1:** HPV detection in prostate and cervical tissue samples from Mexican population.

Sample type	Global prevalence	Genotypes detected	Reference
Benign prostatic hyperplasia and adenocarcinoma	15%	18, 51, 52, 66	Dávila-Rodríguez *et al*., 2016
Prostatitis, normal hyperplasic and carcinoma prostate tissues	13%	33, 45, 52, 58, 66, 68, 83, 44, 81, CP6108	Martínez-Fierro *et al*., 2010
NC, SIL	8%	SI: 59, 51, 45	Jácome-Galarza *et al.,* 2017
		MI: 52-53, 51-59, 61-67, 66-11, 16-62, 53-62, 59-CP6108, 45-66, 45-51	
NC	36%	SI: 51, 52, 16, 33	Gallegos-Bolaños *et al*., 2017
		MI: 16-51, 16-52	
NC, SIL, CT	71%	SI: 16	Romero-Morelos *et al*., 2017
MI:16-52, 16-45			
NC	21%	58	Conde-Ferráez *et al*., 2017
NC, D, CC	20%	59, 52, 16, 56	Fajardo-Ramírez *et al*., 2017
AC	42%	16, 18, 45, 58	González-Losa *et al*., 2017
SIL, CC	91%	SI: 16 58 31 18 70	Ortega-Cervantes *et al*., 2016
		MI: 16-18, 16-51, 16-52, 16-59, 16-66, 16-70	
NC, ASCUS, SIL, CC	68%	33, 16, 18, 51	DelaRosa-Martínez *et al*., 2016
NC, CIN1, CIN3, CC	53%	16, 18, 45, 52, 58, 39, 62, 51, 84, 53, CP6108	Aguilar-Lemarroy *et al*., 2015
NC, AC, CC	18%	16, 58, 52	Magaña-Contreras *et al*., 2015
NC, SIL, CC	67%	SI: 16, 18, 31, 59, 58, 33, 45, 52, 58	Salcedo *et al*., 2014
		MI: 16-31, 16-33 16-45, 16-52, 16-58	
CC	99%	16, 18, 45, 31	Guardado-Estrada *et al*., 2014
NC, SIL, CC	57%	16, 18, 58, 31, 33, 45	Peralta-Rodríguez *et al*., 2012
NC	21%	6 11	Canche *et al*., 2011
NC, SIL	44%	16, 18, 58, 11, 53, 35, 45	Orozco-Colín *et al*., 2010
NC, SIL, CC	80%	16, 33	Illades-Aguiar *et al*., 2010
NC, SIL	31%	16, 18, 31, 6, 11	Velázquez-Márquez *et al*., 2010
NC, SIL, CC	25%	6 11, 16, 18, 31	Velázquez-Márquez *et al*., 2009
SIL, CC	99%	16, 31, 18, 35, 52, 33, 58	López-Revilla *et al*., 2008
NC, SIL, CC	62%	16, 31, 35, 58, 33, 52, 67, 18, 45, 59, 56, 53, 66	Piña-Sánchez *et al*., 2006
SIL, CC	5%	16, 18, 33	Sánchez-Anguiano *et al*., 2006
SIL, CC	56%	58, 16, 18, 33, 31	González-Loza *et al.,* 2004
SIL, CC		16, 18, 33, 35, 58	Montoya-Fuentes et al., 2001
NC, SIL, CC	15%	16, 53, 31, 18	Lazcano-Ponce *et al*., 2001
NC, SIL, CC	55%	16, 18, 45, 39, 59, 58	Torroella-Kouri *et al*., 1998

NC ,normal cytology; SIL, squamous intraepithelial lesions, CC, cervical carcinoma; IC, invasive carcinoma; CT, cervical tumors; D, dysplasia; AC, abnormal cytology; ASCUS, atypical squamous cells of undetermined significance; CIN1, cervical intraepithelial neoplasia grade 1; CIN3, cervical intraepithelial neoplasia grade 3; SI, simple infection; MI, multiple infection.

## Materials and Methods

### Study population

The present study was conducted at the Central Military Hospital of the National Defense Ministry, Mexico City. We studied 356 paraffin-embedded tissue samples from unrelated men over 40 years old, who had undergone radical prostatectomy.

The samples were divided into 2 groups designated controls and cases. The control group comprised 167 benign prostatic hyperplasia (BPH) tissue samples, and the case group comprised 189 tissue samples from men diagnosed with PCa, which was confirmed by histological analysis. The Institutional Human Research Ethical Committee approved the protocol.

### DNA extraction and molecular assays

DNA was extracted from paraffin-embedded tissue samples using the DNeasy Blood and Tissue Kit (QIAGEN Ltd., Crawley, U.K.) according to the manufacturer’s protocol. DNA concentrations were spectrophotometrically determined at 260 nm. The integrity of the DNA samples was assessed by electrophoresis in 1% agarose gels, with the human beta-globin gene being amplified by polymerase chain reaction (PCR) as internal control. HPV detection was performed using consensus primers to amplify part of the L1 gene HPV region, following the previously demonstrated efficacy of PCR amplification from a variety of genital HPV types (Manos *et al.*, 1989). All samples were amplified using three pairs of degenerate primers MY09/MY11, GP5+/6+, and L1C1. The sizes of the amplification products were approximately 450, 150, and 250 bp, respectively. Following PCR for the detection of HPV genotypes, all amplicons were purified using ExoSAP-IT (USB) and sequenced in an ABI PRISM 3130 automated DNA sequencer (Applied Biosystems) using the ABI PRISM BigDye Terminator v3.1 Cycle Sequencing Kit (Applied Biosystems). As Sanger sequencing is not reliable in cases of multiple infection, all HPV-positive samples were also analyzed for the detection of multiple genotypes by means of the MPCR Kit for Human Papilloma Virus Set 2 (Maxim Biotech, Inc). The kit is based on multiplex PCR, which simultaneously amplifies HPV genotypes 6, 11, 16, 18, 31, 33, 52, and 58.

### Histopathological analyses and light microscopy detection of koilocytes in HPV-positive samples

All HPV-positive tissue samples were stained with hematoxylin and eosin (HE). Briefly, the tissues were incubated with Harris’ hematoxylin for 15 min and subsequently washed with distilled water, followed by acid alcohol, running water, and 2% sodium bicarbonate. Subsequently, the samples were fixed in 80% ethanol for 2 min, placed in alcoholic eosin solution for 10 min, and then the samples were decolorized with 90% ethanol to remove excess dye. Finally, the samples were analyzed using a light field optical microscope at 20× magnification.

### *In situ* HPV detection

To identify the high-risk HPV (HR-HPV) (HPV-16, -18, -31, -33, -52b and -58) E6/E7 viral genes, *in situ* PCR was performed using E6/E7 specific primers, as previously described (Fujinaga *et al.*, 1991; Manjarrez *et al.*, 2006). For *in situ* analysis of E6/E7 gene amplification, direct *in situ* PCR was performed as previously described, with some modifications (Nuovo, 2001; Ocadiz-Delgado *et al.*, 2012, 2013). Briefly, dried dewaxed sections on DNase/RNase-free electrocharged slides were incubated with Proteinase K. After thoroughly washing with ultrapure water, PCR optimal solution (master mix) containing digoxigenin-11-(2’-deoxy-uridine-5’)-triphosphate (DIG-11-dUTP; Roche, USA) was added (Nuovo, 2001). Negative controls were generated without a forward primer*. In situ* PCR was performed using a Perkin Elmer system (USA). The amplification of DNA was accomplished using a hot start method with two consensus sequence primer pairs within E6 and E7 of high-risk HPV (pU-1M and pU-2R primers) (Fujinaga *et al.*, 1991) and 5 U of recombinant *Taq* DNA polymerase (Thermo Fisher Scientific, USA). The cycling conditions were 2 min at 94 ºC and 18 cycles of 94 ºC for 1 min, 60 ºC for 1 min and 72 ºC for 1 min. Clips and AmpliCover discs were removed and the slides were washed in PBS, followed by 5 min in 100% EtOH prior to air drying.

### Detection of *in situ* PCR products

We used an indirect immunolabeling method with a primary anti-digoxigenin antibody (Fab fragments; Roche) conjugated to alkaline phosphatase to detect the PCR product. Briefly, blocking was performed in 5% BSA (Sigma, USA) in PBS for 30 min. The slides were subsequently drained and an anti-DIG antibody (diluted 1:200 in 100 mM Tris-HCl, pH 7.4, and 150 mM NaCl) was applied (100 mL per sample) for 2 h at room temperature. The detection of alkaline phosphatase was performed for 10 min using an NBT/BCIP kit (Roche). After detection, the slides were rinsed in distilled water for 5 min and counterstained with Fast Green. The slides were air-dried and subsequently mounted in Permount histological mounting medium (Fisher Scientific, USA).

### Digital image capture and analysis

Images were obtained using a DFC290 HD digital camera (Leica Microsystems, USA). The image files were opened with PhotoImpact software (Ulead PhotoImpact SE ver. 3.02; Ulead Systems, U.S.A.) and digitally processed to obtain a more homogeneous signal.

### Immunohistochemistry

Sections of 5 μm in thickness were obtained from the paraffin blocks and mounted on electrocharged slides. Subsequently, the tissues were de-paraffinized and rehydrated as previously described (Sambrook *et al.*, 1989). Endogenous peroxidase activity was quenched by incubation with 30% hydrogen peroxide in methanol. The sections were washed in PBS (pH 7.4), and nonspecific binding was blocked with 10% bovine serum albumin (Sigma) in PBS for 30 min. Incubation with a monoclonal p16-INK4A antibody (Santa Cruz Biotechnology, Santa Cruz, CA) was performed overnight at 4 °C. Protein detection was performed using the Mouse/Rabbit PolyDetector HRP/DAB Detection System (Bio SB, USA) (Ocadiz-Delgado *et al.*, 2012; Cortés-Malagón *et al.*, 2013). Brown precipitates were observed, indicating the presence of the p16-INKA4 protein.

### Statistical analysis

The chi-squared test was performed, and the odds ratio was determined with 95% confidence intervals using SPSS statistical software, version 17.0 (SPSS Inc., Chicago, IL), as a measure of the association between HPV infection and the risk of PCa.

## Results

In this study, we examined the presence of HPV in paraffin-embedded tissue samples from unrelated Mexican men with PCa or BPH. Using three pairs of degenerate primers targeting the L1 late gene, HPV was detected in 14.9% (53/356) of all tissues analyzed, showing higher levels in tissues with PCa (37/189 or 19.6%) than in tissues with BPH (16/167 or 9.6%), suggesting that HPV infection could be a risk factor for PCa (odds ratio 2.3, 95% CI 1.23–4.3, *p*=0.01).

**Figure 1:**
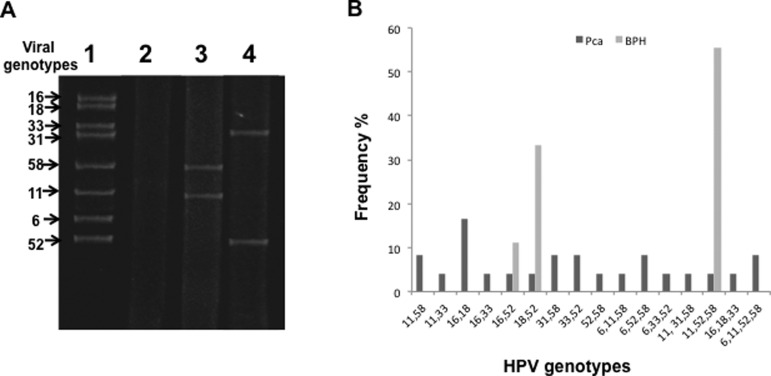
HPV genotypes detected in this study. (A) Electrophoresis of PCR products. Lane 1: positive control (HPV genotypes detected), lane 2: negative control (no added DNA), lanes 3 and 4: representative samples. (B) HPV genotypes frequency in biological samples analyzed.

Genotype detection was evaluated by sequencing from L1 gene amplicons or PCR multiplex. The detection of HR HPV genotypes was 81.4% (83% in the BPH group and 79% in the PCa group), which is much higher than the occurrence of the low-risk (LR) HPV genotypes at 19% (17% in the BPH group and 21% in the PCa group). The viral genotypes observed in the samples in order of decreasing prevalence were HPV 52 (33.3%), HPV 58 (17.17%), HPV 11 (12.7%), HPV 18 (10.8%), HPV 16 (7.8%), HPV 33 (6.9%), HPV 6 (5.9%), and HPV 31 (4.0%) (Table 2). The detection of multiple HPV genotypes in the same sample (2-4 types) was 62.3% (33/53), which was higher than the detection of a single HPV genotype at 37.7% (20/53). Detection of multiple HPV genotypes was not dominant in any of the groups, and both groups showed a high frequency of multiple infection (PCa 65% vs BPH 56.3%). Of the 33 men detected with multiple infection, 19 (57.6%) were co-infected by two types, 12 (36.3%) were co-infected by three types, and 2 (6.1%) were co-infected by four types (Figure 1).

**Table 2:** Frequency of HPV genotypes detected from study samples.

Genotype	Number de samples positive by genotype in CaP group			Number de samples positive by genotype in HPB group			Frequency n (%)
	Simple detection	Multiple detection	Total	Simple detection	Multiple detection	Total	
52	7	10	17	7	9	16	33 (33.3)
58	2	10	12	0	5	5	17 (17.17)
11	0	8	8	0	5	5	13 (12.7)
18	2	6	8	0	3	3	11 (10.8)
16	0	7	7	0	1	1	8 (7.8)
33	1	6	7	0	0	0	7 (6.9)
6	0	6	6	0	0	0	6 (5.9)
31	1	3	4	0	0	0	4 (4.0)

With respect to HPV genotype distribution in the study groups, in HPV infections involving a single genotype, HPV 52 was the most common genotype found in both groups, while in HPV infections with multiple genotypes, the most common genotypes were HPV 58 and 52 for the PCa group, and HPV 52, followed by HPV 11 and 58, for the control group (Table 1). The most frequent combinations of genotypes detected were 16/18 for the PCa group and 11/52/58 for the BPH group (Figure 1).

**Figure 2:**
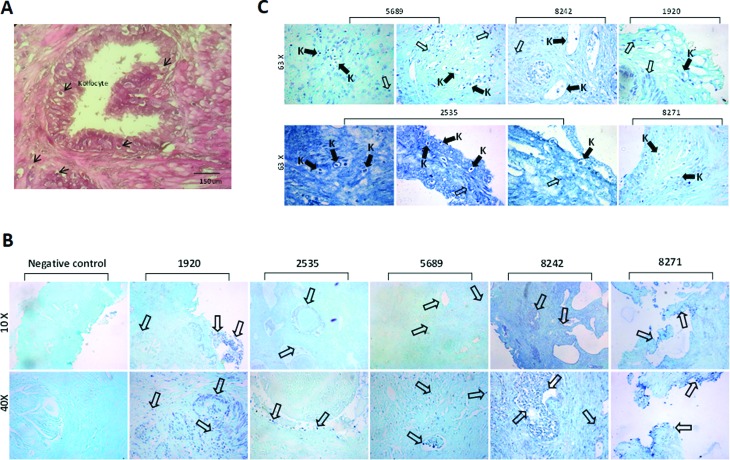
Histopathological and molecular analysis of prostate cancer tissues. (A) Koilocytes were observed in several HPV-infected prostate cancer tissues. Arrows indicate the koilocytes in a representative image. (B) *In situ* HR-HPV detection. HR-HPV E6/E7 DNA was detected in prostate cancer sections employing *in situ* PCR as indicated in the Materials and Methods. The signal was mainly nuclear (indicated by empty arrows). Magnification: 10× and 40×. (C) Solid arrows indicate a positive signal of HPV DNA amplification in koilocytes (K). Magnification: 63×. The numbers indicate the control number of each patient. Negative control: no forward primers were added for *in situ* PCR.

**Figure 3:**
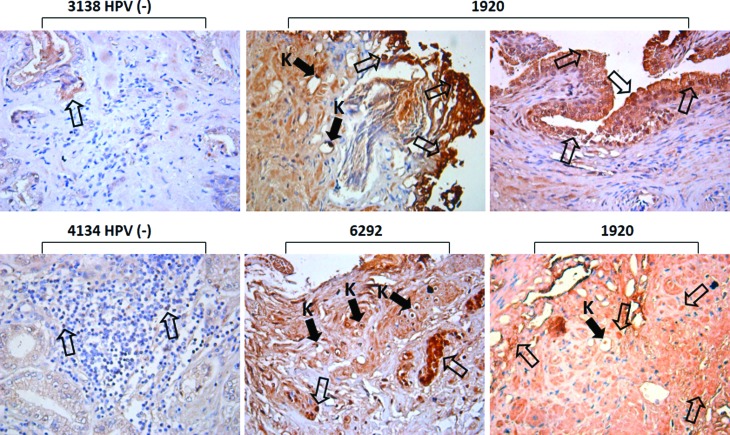
Immunohistochemical detection of p16INK4A in prostate cancer. A strong positive (empty arrows) signal of p16INK4A protein was detected in HPV-positive tissues compared with HPV-negative [HPV(-)] tissues. Solid arrows indicate a positive signal of p16INK4A in koilocytes (K). Magnification: 40×. The numbers indicate the control number of each patient.

Additionally, we identified koilocytes, cells containing an acentric, hyperchromatic nucleus displaced by a large perinuclear vacuole. Although the genesis of the cytoplasmic vacuole remained unclear, particularly because both HPV DNA replication and virion assembly exclusively occur in the nucleus, in clinical biopsies from cervical cells, koilocytosis is observed in both LR and HR HPV infections (Krawczyk *et al.*, 2008). Therefore, in this study, we demonstrated that all HPV-positive samples showed koilocytosis. (Figure 2). Moreover, by using immunohistochemical assays, p16-INK4A protein overexpression was demonstrated in HPV-positive PCa tissue, indirectly suggesting the presence of HR-HPV E7 oncoprotein (Figure 3). As expected, HPV-negative tissue showed low levels p16-INK4A protein expression (Figure 3).

## Discussion

In this study, we examined the presence of HPV in two study groups: a control group comprising BPH tissues and a case group comprising cancerous tissues. We found HPV in 53 (14.9%) of the 356 tissues analyzed, which is similar to the findings in other reports of tissue analysis from Latin America (18.63%) (Yang *et al.*, 2015) and Mexico (11.5%-14.9%) (Martínez-Fierro *et al.*, 2010; Dávila-Rodríguez *et al.*, 2016). Consistent with the meta-analysis and other reports from Mexico, in the present study, we detected an association between HPV and PCa, with different frequencies of HPV in the two study groups. It is important to consider that because it is difficult to obtain normal prostate tissues, in this study, we used BPH tissues as controls; however, the use of normal prostate tissues as controls may show a higher association between HPV and PCa.

Some reports have described a role for HPV in PCa, suggesting that infection triggers chronic recurrent inflammation, and the prostate gland could be infected owing to its anatomic proximity to the anogenital and urinary sites, thus being in support of the association of cancer with HPV (Rabkin *et al.*, 1992; Guma *et al.*, 2016; Tolstov *et al.*, 2014). The detection of HR genotypes was much higher than that of LR genotypes, which confirms the frequent identification of HR HPVs in PCa seen in many studies (Bae, 2015; Yang *et al.*, 2015).

In contrast to other reports showing that the HPV types 16 and 18 are the most prevalent, in this work, the most prevalent HPV types were 52 and 58 in both study groups. These differences may be related to the fact that the distribution of HPV varies among different populations, as has been well recognized in previous epidemiological studies (Bae, 2015; Yang *et al.*, 2015). Moreover, the genotypes detected in our prostate tissue samples are consistent with previous reports from Mexican populations (Martínez-Fierro *et al.*, 2010; Dávila-Rodríguez *et al.*, 2016), and these genotypes are also prevalent in cervical cancer samples from different areas of Mexico, as summarized in Table 1, demonstrating the importance of sexual transmission as a route for dissemination of the virus.

Some researchers have reported that co-infections in cervical cells may be associated with higher persistence rates of certain HR-HPV types compared with those of LR-HPV types or single infection (Trottier *et al.*, 2006; De Brot *et al.*, 2017). In this study, the detection of HR-HPV genotypes was much higher than that of LR-HPV genotypes in both groups; however, the most frequent combinations of genotypes detected were 16/18 and 11/52/58 for PCa and BPH, respectively. Therefore, evidence suggests that BPH with HR HPV co-infections could be a precursor of PCa, consistent with chronic recurrent inflammation as a known cause of PCa (De Marzo *et al.*, 2007; Elkahwaji, 2012; Sfanos *et al.*, 2014).

A long-recognized, pathognomonic feature of HPV infection is the appearance of halo or koilocytotic cells. Here, we detected koilocytes, which have been identified in prostate tissues in other studies (Whitaker *et al.*, 2013). Koilocytes were detected in 100% of the samples with HPV. Moreover, the *in situ* PCR detection of HR-HPVE6E7 genes and p16-INK4A overexpression in PCa tissues similar to that in human prostate epithelial cell lines (Ko *et al.*, 2003; Theodore *et al.*, 2010) and a male case of urothelial carcinoma with squamous differentiation associated withHPV in another report (Guma *et al.*, 2016) are suggestive of early and late ongoing oncogenic processes in BHP and PCa, respectively. This hypothesis is supported by a recent meta-analysis that demonstrated that BPH was associated with an increased incidence of PCa (Dai *et al.*, 2016) and the fact that HR-HPVs have been identified in both benign and malignant prostate tissues (Lin *et al.*, 2011; Bae, 2015; Yang *et al.*, 2015). Moreover, recent evidence has shown that in Australian men, HR-HPVs are present in benign prostate tissues before the development of HPV-associated PCa (Glenn *et al.*, 2017).

The results presented here are important for the following reasons: (i) development of diagnostic assays; (ii) evaluation of the impact of vaccination in cancer prevention strategies, especially since many HPV genotypes are not covered by the current quadrivalent HPV vaccine used in Mexico; and (iii) description of highly prevalent genitourinary tract HPV infections in sexually active men in México (Lajous *et al.*, 2005; Giuliano *et al.*, 2008; Méndez-Martínez *et al.*, 2014) that may be important reservoirs of persistent HPV and play an important role in the pathogenesis and progression of BPH and PCa (Gandaglia *et al.*, 2017).

In conclusion, the high frequency of detection of HPV in PCa, combinations of genotypes with oncogenic potential that dominated in the PCa group, identification of HPV associated koilocytes, and overexpression of p16INK4A in prostate cancer specimens constitute evidence suggesting the association of HPV with PCa and a potential role for the virus in the etiology of PCa.
